# Phylogeographic and Genomic Insights Unveil the Evolutionary History and Post‐Glacial Recolonization Routes of the Palmate Newt (*Lissotriton helveticus*) Into Europe

**DOI:** 10.1002/ece3.71994

**Published:** 2025-09-03

**Authors:** Bernat Burriel‐Carranza, Jhulyana López‐Caro, Adrià Jordà, Loukia Spilani, Adrián Talavera, Gabriel Mochales‐Riaño, Martiño Cabana, Albert Montori, Pierre‐André Crochet, Ernesto Recuero, Mario García‐París, Íñigo Martínez‐Solano, Daniel Fernandez‐Guiberteau, Salvador Carranza

**Affiliations:** ^1^ Museu de Ciències Naturals de Barcelona P° Picasso s/n Barcelona Spain; ^2^ Institute of Evolutionary Biology (CSIC‐Universitat Pompeu Fabra) Barcelona Spain; ^3^ Centre de Recerca i Educació Ambiental de Calafell (CREAC‐GRENP‐Ajuntament de Calafell) Tarragona Spain; ^4^ Facultat de Biologia Universitat de Barcelona Barcelona Spain; ^5^ Grupo de Investigación en Bioloxía Evolutiva (GIBE), Departamento de Bioloxía, Facultade de Ciencias Universidade da Coruña A Coruña Spain; ^6^ CEFE, CNRS Univ Montpellier, EPHE, IRD Montpellier France; ^7^ (MNCN) Museo Nacional de Ciencias Naturales MNCN‐CSIC Madrid Spain

**Keywords:** amphibians, biogeography, dispersal, genomics, historical demography, last glacial maximum, *Lissotriton helveticus*, palmate newt, phylogeography, SNPs

## Abstract

Quaternary glacial cycles have been key drivers of diversification for Holarctic species, promoting divergence, isolation, and extinction processes in numerous taxa. These cycles facilitated evolutionary radiations in some groups but also erased much of the evolutionary history of species with northern origins. Here, we investigate the evolutionary and phylogeographic history of the Palmate Newt ( *Lissotriton helveticus* ), a widespread species in post‐glacial ecosystems in Western Europe. We generate genome‐wide ddRADseq for 205 individuals from 51 populations across the species range and reconstruct its phylogeographic and demographic history, assess population structure, and characterize ecological paleoniches for the species at different climatic periods. Results identify several distinct lineages exhibiting strong genetic differentiation, primarily driven by geographic barriers and isolation in historical refugia with admixture in transition zones. Phylogeographic reconstructions suggest that the main glacial refugium for *L. helveticus* was most likely located in northern Iberia. Two main dispersal routes were identified: one extending eastward through the Ebro River Basin and a second, following a northeastward pathway across the Pyrenees and into Europe. We specifically pinpoint the origin of Europe's recolonization route to a specific set of localities surrounding Andorra, where *L. helveticus* probably expanded along tributaries to the Garonne River into southern and western France over warm periods. By integrating genomic, geographic, and paleoclimatic data, this study provides an in‐depth understanding of how climate shaped the evolutionary history of this temperate species and reinforces the importance of waterways for amphibian dispersal dynamics.

## Graphical abstract

This study investigates the evolutionary and biogeographic history of the palmate newt ( *Lissotriton helveticus* ) by generating a genomic dataset from over 200 specimens across the entire distribution range of the species.

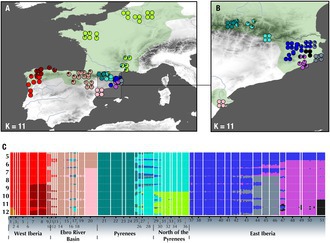

## 1 Introduction

Quaternary glacial cycles acted as major drivers of the diversity, distribution, and evolutionary history of numerous Palearctic taxa. The rapid succession of glacial and interglacial periods promoted cycles of local extinction, isolation, and diversification during glacial maxima, followed by population expansion and secondary contact during interglacials (Babik et al. 2005; Martínez‐Solano et al. 2006; Willis et al. 2004). The progressive cooling since the Pliocene, which eventually led to major expansions of ice sheets across most of northern Europe during Quaternary glacial maxima, forced many species southward into climatic refugia within the Mediterranean penínsulas and around the Mediterranean Basin. These refugia not only safeguarded native taxa but also provided shelter for more northerly distributed species, increasing regional biodiversity in southern regions through lineage accumulation.

For taxa originating in southern European peninsulas, long‐term isolation often promoted divergence and speciation, involving the persistence of several ancient lineages, according to the “refugia within refugia” paradigm. In contrast, northern‐origin taxa that survived glacial periods by shifting their distribution range to southern refugia often experienced strong genetic bottlenecks, retaining only the fraction of their ancestral genetic diversity represented in the populations and lineages that dispersed into climatically favorable regions (Recuero and García‐París 2011). These contrasting demographic histories leave distinct genomic signatures, which led Recuero and García‐París (2011) to propose two biogeographic models shaping these patterns. The so‐called “refuge” model represents species whose ranges shift to more southerly and more restricted climatically suitable areas during glacial maxima, often resulting in reduced genetic diversity and shallow intraspecific differentiation. In contrast, the “sanctuary” model, describes native species persisting within their original range in fragmented but climatically stable refugia, fostering higher genetic diversity and deeper population and lineage divergence events, generally predating the glacial maxima. In the Iberian Peninsula, examples of Plio‐Pleistocene divergences associated with the “sanctuary” model include amphibians like the fire salamander *Salamandra salamandra* (Burgon et al. 2021; Gippner et al. 2024), the Iberian Newt *Lissotriton boscai* (Martínez‐Solano et al. 2006), the Pyrenean brook newt *Calotriton asper* (Lucati et al. 2020; Talavera et al. 2024), the Marbled Newt *Triturus marmoratus* (Kazilas et al. 2024), and midwife toads of the genus *Alytes* (Ambu et al. 2023; Dufresnes and Hernandez 2021; Dufresnes and Martínez‐Solano 2020; Martínez‐Solano et al. 2004). In contrast, the shallow genetic differentiation found in *Vipera seoanei* (Martínez‐Freiría et al. 2015; Talavera, Palmada‐Flores, et al. 2025) or the palmate newt *Lissotriton helveticus* (Recuero and García‐París 2011) illustrate the “refuge” model. Notably, although glacial refugia in southern European peninsulas greatly shaped the demographic history and genetic structure of these taxa, post‐glacial recolonization of Europe appears to have proceeded independently of the underlying biogeographic model, with *Salamandra salamandra* , *Alytes obstetricans,* and *Lissotriton helveticus* currently occupying most of central Europe.

The genus *Lissotriton* illustrates both biogeographic models. This ancient group of salamandrid newts, which originated in Europe approximately 55 million years ago (Ma; Zhang et al. 2008), was largely restricted to the Mediterranean peninsulas during the Quaternary glaciations, serving as both refugia and sanctuaries for different species. Southern‐origin species, such as *L. boscai* , *L. vulgaris* , and *L. italicus* , retained Plio‐Pleistocene mitochondrial lineages through the Quaternary (Canestrelli et al. 2012; Martínez‐Solano et al. 2006; Pabijan et al. 2015). Some of these deeply differentiated lineages in *L. boscai* and *L. vulgaris* were even granted species status, including *L. maltzani* and *L. graecus* , respectively (Pabijan et al. 2015, 2017; but see also Mars et al. 2025). In contrast, species with more northern origins, like the palmate newt *Lissotriton helveticus*, are largely depleted of genetic diversity. *Lissotriton helveticus* occurs widely from the northern half of the Iberian Peninsula to the Czech Republic, including populations across most of Great Britain. Despite originating approximately 20 Ma (Mars et al. 2025; Recuero and García‐París 2011), intraspecific lineage divergence is shallow (~1 Ma). Previous studies on the basis of a few mitochondrial and nuclear markers have postulated a Central European origin of the species, with subsequent colonization of the Iberian Peninsula during the Pleistocene, following global climate cooling events. Thus, during the Quaternary glacial periods, *L. helveticus* was likely extirpated from most of its native range, resulting in the loss of deeply differentiated lineages that did not reach the Iberian Peninsula (Recuero and García‐París 2011). In the Iberian Peninsula, four major mitochondrial DNA haplogroups originated within the last million years, distributed along a west‐to‐east geographic cline (Recuero and García‐París 2011).

Reconstructing phylogeographic histories in species following the “refuge” model remains challenging, given their overall low levels of genetic diversity and shallow genetic structure, which complicate robust inferences of lineage relationships and historical dispersal routes. For instance, the current distribution of the species across Europe appears to result from a relatively recent expansion, with all sampled populations sharing a single mitochondrial haplotype in the Cytochrome oxidase subunit 1 (COI) gene (Elfering et al. 2024). This lack of genetic variation has hindered accurate reconstructions of the out‐of‐Iberia recolonization route (Elfering et al. 2024). In this context, genome‐wide markers such as SNPs from ddRADseq genomic libraries can provide higher resolution, allowing for a more detailed analysis of the complex and recent evolutionary history of this temperate species.

In the present study, we assemble a comprehensive genomic ddRADseq dataset from 205 individuals sampled in 51 localities covering most of the distribution range of *L. helveticus* , with a special focus on the Iberian Peninsula. We perform an integrative approach to interrogate the diversification dynamics and the evolutionary and demographic history of *L. helveticus* , and reconstruct the phylogeographic history and ecological paleoniches of the species to determine the most plausible recolonization route into Europe. Additionally, we reconstruct a genomic phylogeny with several representatives of the genus to determine the phylogenetic relationships between *Lissotriton* species.

## 2 Materials and Methods

### 2.1 Sampling, Sequencing, and Dataset Creation

**FIGURE 1:**
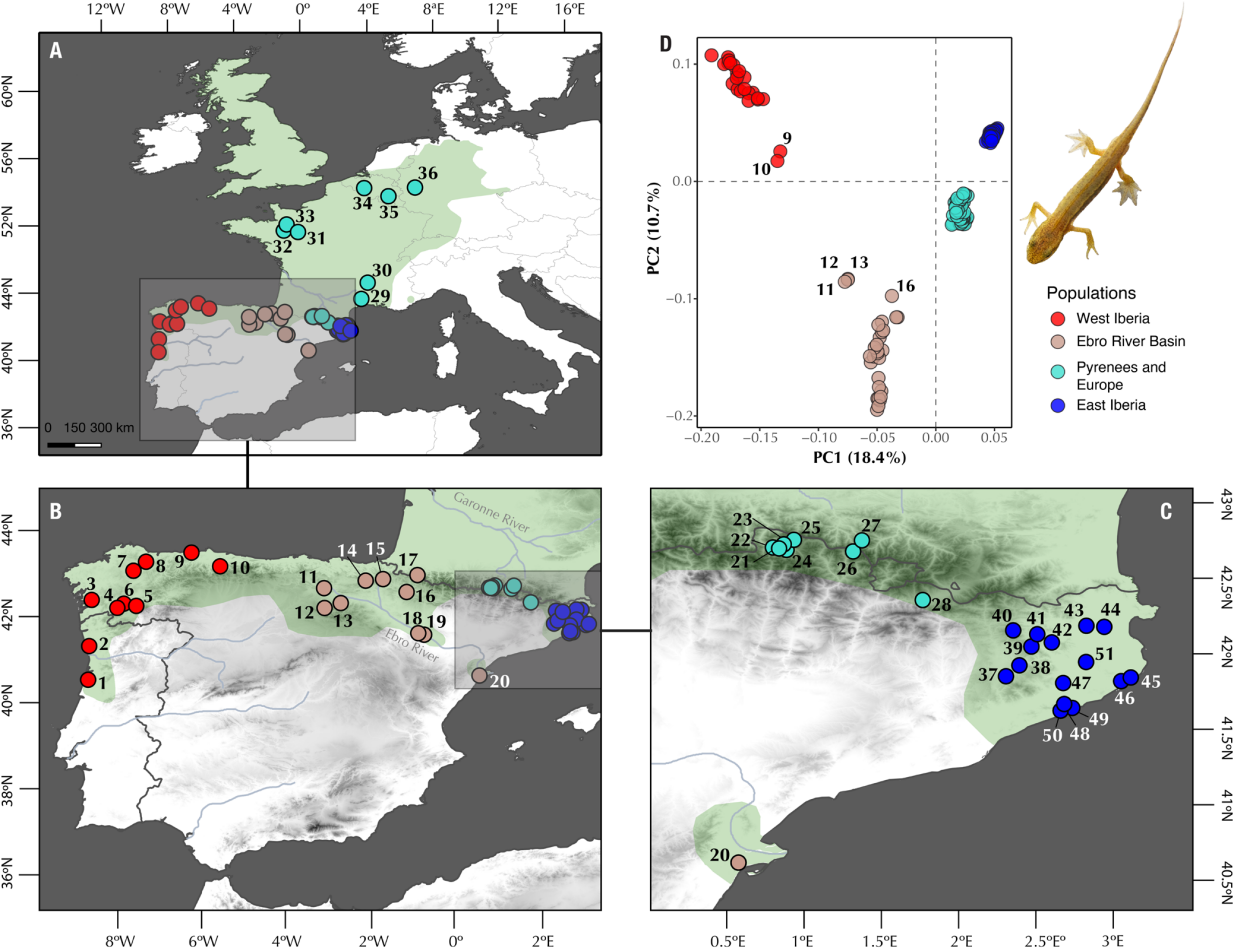
Distribution range of *Lissotriton helveticus* (A–C) displaying 51 sampling localities identified by numbers (see Table S1 for coordinates). Colors represent genetic clusters on the basis of PCA results (see text for details). The species distribution map was adapted from the IUCN Red List of Threatened Species (www.iucnredlist.org). (D) Principal component analysis (PCA) derived from 3603 putatively unlinked SNPs (uSNPs), showing population structure in *L. helveticus* . Numbers in caption D refer to locality codes discussed in the text.

We sampled a total of 205 *Lissotriton helveticus* individuals from 51 different localities (1 to 20 individuals per locality), mostly collected between 2018 and 2019, but also including some specimens analyzed by Recuero and García‐París (2011). Tissue samples were obtained from tail tips and stored in 99% ethanol until DNA extraction. The sampling encompassed the entire species distribution range, with a primary focus on the Iberian Peninsula, particularly in Catalonia (Figure 1; Table S1). To infer the phylogenetic relationships of *L. helveticus* to other species within the genus, we also obtained eight samples from four *Lissotriton* species (three *L. italicus* , two *L. boscai*, and three *L. vulgaris* ) and two *Triturus marmoratus* that were used as outgroup.

We generated double‐digest restriction‐site associated DNA (ddRAD) libraries with the protocol implemented in Peterson et al. (2012) using a combination of rare and common restriction enzymes (Sbf1 and Msp1, respectively). Fragments were purified with Agencourt AMPure beads before ligation with eight barcoded Illumina adapters. Samples with unique adapters were pooled, and each eight‐sample pool was size‐selected for a distribution of 500 bp fragments using BluePippin (Sage Science). Illumina multiplexing indices were ligated to individual samples using a Phusion polymerase kit (high‐fidelity Taq polymerase, New England Biolabs) and sequenced on an Illumina NexSeq 500 with 75 bp single‐end reads. Sequence data are available under the BioProject PRJNA1241519 stored in GenBank.

Raw Illumina reads were processed using iPyRAD v.0.7.8 (Eaton and Overcast 2020). Sites with Phred quality scores below 99% (Phred score = 33) were flagged as missing data. Then, reads with three or more missing sites, consensus sequences with fewer than 10 reads, or more than two alleles were discarded. We also filtered by maximum read depth and excessive heterozygous sites (more than three) to discard potential paralogous loci. Upon testing several configurations, both filtered reads and consensus sequences were clustered and aligned using an 89% clustering threshold. The minimum number of samples per locus was set to four. An additional filtering step was then applied to discard iteratively and alternatively low‐quality samples and genotypes (Burriel‐Carranza et al. 2023). Missing data allowance for both samples and genotypes was iteratively filtered from 98% to 60%, decreasing by 2% between iterations. A hard threshold for missing genotype call rate was then applied, depending on the dataset, to minimize the overall missing data without discarding additional specimens. Finally, we removed all non‐biallelic SNPs, applied a minor frequency filter (maf < 0.05), and discarded monomorphic sites. For the different analyses, we retrieved *loci*, *haplotype*, and *SNP* data depending on the analyses' requirements.

*Loci* datasets were obtained by implementing a second round of Ipyrad, keeping only those specimens that passed the previous filters and retaining all variant and invariant sites. For population structure analyses and SNAPP, we generated a *SNP* dataset by keeping putatively unlinked SNPs (uSNPs), selecting one SNP with the highest depth per locus. If there were more than one SNP with the highest depth, one was chosen at random.

### 2.2 Phylogenomic and Phylogeographic Reconstructions

We reconstructed phylogenomic relationships in *Lissotriton* by means of maximum likelihood (ML) and calibrated Bayesian Inference (BI) approaches. First, we generated a *Loci* dataset containing 13 specimens (i.e., representatives of *L. helveticus, L. boscai, L. vulgaris, L. italicus*, and *Triturus marmoratus* as outgroup), and 1941 loci allowing for a maximum of 40% missing data per locus (see Table S1, Dataset 1 for details on the selected specimens). We concatenated all loci to generate ML reconstructions with RAxML‐ng v.1.0.2 (Kozlov et al. 2019), using a GTR + G model, a total of 100 starting trees (50 random and 50 parsimony) and 1000 bootstrap replicates to evaluate branch support. Second, we used the same specimens to infer a coalescent‐based time‐calibrated species tree inferred with SNAPP implemented in BEAST2 v2.6.4 (Bryant et al. 2012). To do so, we generated a dataset of 1059 unlinked SNPs and allowed a maximum of 10% missing data at each locus and used the “snap_prep.rb” script (https://github.com/mmatschiner/snapp_prep) to calibrate the phylogeny dating its deepest node (the split between *Triturus* and *Lissotriton*), applying a lognormal distribution from a mean age of 50.43 Ma and a *σ* of 7.2 (Recuero and García‐París 2011; Wiens et al. 2011; Zhang et al. 2008). Three independent runs were conducted for 5 × 10^6^ generations, sampling every 2500 generations. Convergence between runs and effective sample size of the parameters posterior probability (ESS > 200) were checked with Tracer v1.7.0 (Rambaut et al. 2018). After visualization, a 20% burn‐in was applied, and runs were combined and annotated with LogCombiner v2.6.4 and TreeAnnotator v2.6.4 (Bouckaert et al. 2019), respectively.

We conducted further ML and BI phylogenomic analyses in *L. helveticus* , using three *L. vulgaris* individuals as outgroups. First, we selected up to two individuals per locality (those with the highest coverage) to generate a dataset of 93 individuals (90 *L. helveticus* and 3 *L. vulgaris* ). Then, we generated a *loci* dataset retaining all loci present in at least 60% of all the samples, recovering a dataset of 16,337 loci and 54,946 SNPs, with an overall dataset missingness of 23.85% (see Table S1, Dataset 2 for details on the selected specimens). We concatenated all loci and generated ML reconstructions with RAxML‐ng (Kozlov et al. 2019), following the same specifications as described above. A multispecies‐coalescent time‐calibrated species tree was also inferred with SNAPP implemented in BEAST2 v2.6.4 (Bryant et al. 2012) using a reduced dataset of 26 specimens, 1870 uSNPs, and a maximum missing data allowance of 10%. A total of 15 taxa (14 *L. helveticus* populations and *L. vulgaris* ) were defined on the basis of the best population configuration results in ADMIXTURE analyses (see below) and by acknowledging political borders. Up to two specimens from each population were then selected, prioritizing those with the highest coverage (see Table S1, Dataset 2 for details on the selected specimens). Additionally, admixed individuals were excluded to avoid model violations (i.e., SNAPP assumes no gene flow between populations). We then used the age estimate of the split between *L. vulgaris* and *L. helveticus* obtained in our time‐calibrated phylogeny reconstructed in this study to date the deepest node in this analysis with a lognormal distribution of 25.55 my and a *σ* of 4.9 with the script “snap_prep.r”. Three independent runs were conducted for 5 million generations, sampling every 2500 generations. Convergence between runs and ESS were ensured with Tracer v1.7.0 (Rambaut et al. 2018), and a 10% burn‐in was applied. Runs were combined with LogCombiner v2.6.4, and annotated with TreeAnnotator v2.6.4 (Bouckaert et al. 2019).

To infer the phylogeographic history of *L. helveticus* and reconstruct the most plausible origin for the out‐of‐Iberia recolonization, we performed an ancestral range reconstruction by means of BioGeoBears (Matzke 2018). On the basis of population structure results (see below), we divided the species range into five regions: West Iberia, Ebro River Basin, Pyrenees, East Iberia, and North of the Pyrenees. We assigned each tip of the reconstructed species tree to its respective range, set the maximum number of areas per node to two, and performed ancestral reconstructions using the following models: Dispersal‐Extinction‐Cladogenesis (DEC; Ree and Smith 2008), DIVALIKE (Ronquist 1997) and BAYEAREA (Landis et al. 2013). Then, we selected the best model (DIVALIKE) on the basis of the Akaike Information Criterion correcting for small sample size (AICc; Akaike et al. 1973).

### 2.3 Population Structure and Genetic Diversity

We inferred the population structure of *Lissotriton helveticus* by means of a principal component analysis (PCA) and ADMIXTURE v1.3 (Alexander et al. 2009; Alexander and Lange 2011). A total of 205 specimens were included in these analyses, and the final unlinked SNP data assembly allowed a maximum of 40% missingness at each locus (see Table S1, Dataset 3 for details on the selected specimens). The final dataset contained 3603 uSNPs and an overall dataset missingness of 4.8%. The PCA was conducted with Plink v1.90 (Chang et al. 2015). We then evaluated a range of possible populations (*K*) for *L. helveticus* in ADMIXTURE v1.3 (Bouckaert et al. 2019) ranging between 1 to 20, with 15 replicates and 15 cross‐validation (CV) rounds. Best K values were chosen on the basis of CV scores, as well as on their plateau profiles and the likelihood of the result in biological terms. We also characterized genetic diferentiation across *L. helveticus* populations by assessing population‐pairwise standardized fixation indexes (F'_ST_). We used the uSNPs dataset of 205 individuals and 3603 uSNPs (see details above) to calculate population‐pairwise F'_ST_ using GenoDive v.3.0 (Meirmans 2020). Localities were assigned to the 11 populations identified in the admixture analyses with the exception of the populations from the Ebro River Basin, which were further split into localities 11–13, 14–17, and 18–19, and populations from Europe, which were further divided into localities 29–30, 32–33, 34–35, and 36.

To explore the differences in population structure across *L. helveticus* lineages, we tested for isolation‐by‐distance (IBD). These IBD tests examine whether the genetic distances between populations belonging to two distinct lineages are not larger than expected on the basis of their geographic distances and within‐lineage IBD patterns (Bamberger et al. 2022; Hausdorf and Hennig 2020). To estimate geographic distances between populations, Euclidean distances (in kilometers) were computed and subsequently log‐transformed. Genetic distances were quantified as F'_ST_/(1‐F'_ST_) (Weir and Cockerham 1984). The R package *prabclus* (Hennig and Hausdorf 2019) was used to generate a Jackknife‐based test, assessing whether IBD patterns were comparable within each pair of metapopulations. If the within‐lineage IBD signal regressions between assessed lineages evolved similarly (non‐significant differences observed between regressions), both were merged into a single joint within‐lineages regression. A second Jackknife‐based test was then conducted to compare the joint regression with another including all within‐ and between‐lineage comparisons. If these two regressions were significantly different, it would indicate that genetic differentiation between lineages cannot be solely explained by IBD.

### 2.4 Demographic History

To reconstruct the demographic history of *L. helveticus,* we first generated a *SNP* dataset containing 205 *L. helveticus*, retaining all SNPs present in at least 60% of the samples (see Table S1, Dataset 3 for details on the selected specimens). In this analysis, we did not apply the maf filter described above. Then, we downprojected the dataset by means of the easySFS script (https://github.com/isaacovercast/easySFS) into a Site Frequency Spectrum (SFS), considering all individuals as belonging to the same deme. We chose the optimum number of retained haploid sequences without missing data on the basis of the number of segregation points (107 sequences, 80,530 segregation points). We then used the intraspecific folded SFS file to generate a blueprint for Stairway Plot v.2.1.1 (Liu and Fu 2020), assuming a 4‐year generation time and a mutational rate of 7.7 × 10^−9^ (Wu et al. 2013). The total number of sites was estimated from the percentage of monomorphic sites in the species. Median effective population size (Ne) estimates were based on 200 runs and were plotted along with their respective 95% confidence intervals. The results of the demographic inference were then visualized along Northern Hemisphere continental mean surface air temperature anomalies for the last 10^5^ years (de Boer et al. 2014). We statistically evaluated the correlation between variables (Ne and temperature anomalies) by fitting a generalized least squares (GLS) model over 10,000 permutations using a randomized residual procedure in the R package “RRPP” v. 1.3.1 (Collyer and Adams 2023; Collyer and Adams 2018). To account for temporal autocorrelation in the GLS, we built a covariance matrix incorporating the decay of temporal autocorrelation from an autoregressive model (AR1) implemented in the R package “nlme” v.3.1–162 (Pinheiro 2011).

### 2.5 Ecological Niche Modeling (ENM)

To understand the effects of Pleistocene climatic oscillations on habitat suitability and historical dispersal dynamics in *L. helveticus* , we used ENM creating models for current climatic conditions and projecting them to a series of past paleoclimatic reconstructions. We compiled a dataset of 4241 occurrence records spanning the entire *L. helveticus* distribution from unobscured, research‐grade, iNaturalist records (https://www.inaturalist.org) with a positional accuracy of at least 4 km. To ensure spatial homogeneity and minimize spatial bias (Merow et al. 2013; Sillero et al. 2021), records were thinned to one per 20 km, resulting in a dataset of 788 occurrences.

For current climatic conditions, we downloaded 19 climatic variables at 2.5 arc minutes (~5 × 5 km) from WorldClim (Hijmans et al. 2005). We tested spatial correlation among variables and, to avoid collinearity in the predictors used to train the ecological models, retained four low‐correlated variables (*R* < 0.6) with biological relevance for the study species (Bio1, Bio12, Bio14, and Bio15). For paleoclimatic conditions, we downloaded the same four bioclimatic variables at 2.5 arc minute (~5 km) resolution from Paleoclim (http://www.paleoclim.org/) for the following periods: Mid‐Pliocene Warm Period (MPWP; 3.3 Ma); Last Interglacial (LIG; 130 kya); Last Glacial Maximum (LGM; ~21 kya); Heinrich Stadial 1 (HS1; 17–14.7 kya); Bølling‐Allerød (BA; 14.7–12.9 kya); Younger Dryas Stadial (YDS; 12.9–11.7 kya); early‐Holocene, Greenlandian (EHG; 11.7–8.326 kya); and mid‐Holocene, Northgrippian (MH; 8.326–4.2 kya). Each paleoclimatic layer was masked and cropped to the geographic extent encompassed by the countries where *L. helveticus* occurs.

To determine climatic requirements and paleoclimatic responses of *L. helveticus* we used the biomod2 package (Thuiller et al. 2009) implemented in R. To avoid spatial biases in the models, the area used for training was defined according to the known distribution of *L. helveticus* (https://www.iucnredlist.org/), with a buffer of 100 km (Sillero et al. 2021). Pseudo‐absences were then generated using a disk strategy with minimum distances of 50 km from occurrence points. We created 10 replicates of 5000 pseudo‐absence points to ensure robust model performance. Models were developed using Generalized Linear Models (GLM) with a 10‐fold cross‐validation strategy, partitioning the data into 80% training and 20% testing subsets. Model robustness was evaluated across 10 replicates, producing 100 models. We assessed model performance using the True Skill Statistic (TSS), a threshold‐independent performance metric, and the area under the curve (AUC) of the receiver operator characteristic plot. We also estimated variable importance scores to assess the relative contribution of each environmental predictor. Model replicates were developed under the training area and then projected to the entire study area for current and past climatic conditions. All model replicates and projections with TSS scores above 0.6 were used to derive ensemble models and projections. Uncertainty of each ensemble model was assessed by constructing the coefficient of variation maps.

## 3 Results

### 3.1 Phylogenomic Analysis

#### 3.1.1 Phylogenetic Analysis of the Genus *Lissotriton*

**FIGURE 2:**
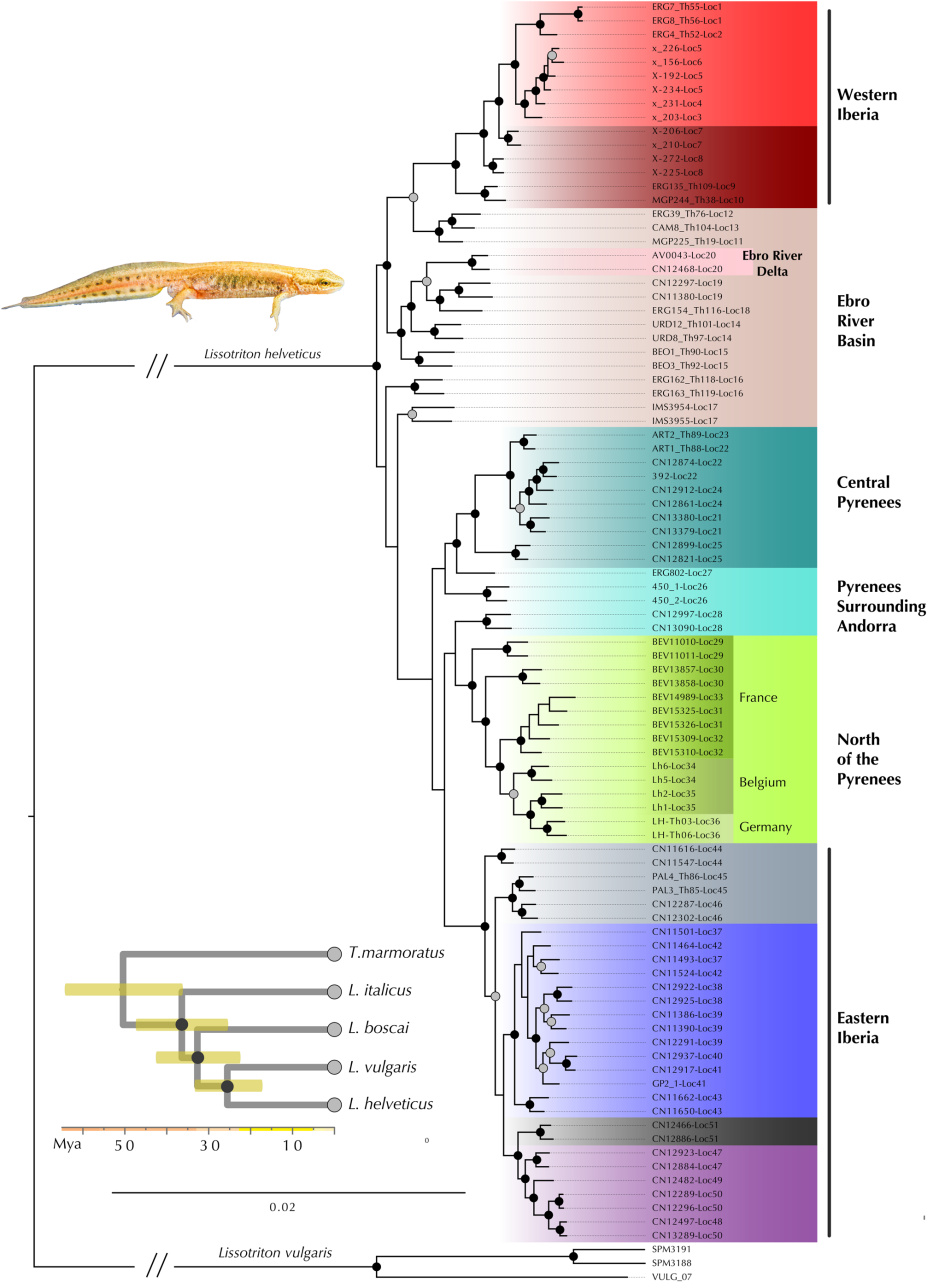
Maximum likelihood phylogenomic reconstruction of intraspecific relationships within *Lissotriton helveticus* . Groups are color‐coded on the basis of the assignment of each individual to the corresponding ADMIXTURE cluster. The inset displays a time‐calibrated species tree including *Triturus marmoratus* and four *Lissotriton* species as sequential outgroups. Bootstrap support values of 100 and posterior probabilities of 1 are represented by black circles. Gray circles represent bootstrap support above 85.

All our phylogenomic tree reconstructions (ML and BI) recovered *L. vulgaris* as the sister taxon to *L. helveticus* (our sampling did not include *L. montandoni* , identified by Mars et al. (2025) as the closest relative to *L. vulgaris* ), with an estimated divergence time of 25.6 Ma (95% highest posterior density interval [HPDi]: 17.4–33.1 Ma). The oldest divergence in the genus corresponds to the split between *L. italicus* and all other analyzed *Lissotriton* species about 36.41 Ma. Additionally, *L. boscai* is inferred to have diverged from *L. vulgaris* and *L. helveticus* around 32.7 Ma (Figure 2).

#### 3.1.2 Phylogenomic and Phylogeographic Reconstructions in *Lissotriton helveticus*

The ML phylogenomic reconstruction of *Lissotriton helveticus* reveals complex patterns of genetic structuring that generally align with the species' geographic distribution. Populations are grouped into several lineages corresponding to geographic regions, but their relationships are unclear, with weak support for some nodes suggesting the influence of secondary contacts and admixture (Figure 2).

The first intraspecific split separates West Iberia + Ebro River Basin populations from a Northeastern clade encompassing Pyrenean, Eastern Iberian, and European populations. Within the West Iberia + Ebro River Basin clade, West Iberian localities (localities 1–10) form a well‐supported monophyletic group, sister to localities distributed across the Ebro River Basin (localities 14, 15, 18–20). The Ebro River Delta population is nested within the Ebro River Basin clade, as sister to the specimens from localities 18 and 19. Interestingly, localities 11, 12, and 13, although geographically part of the Ebro River Basin group, cluster with populations from West Iberia, although with weak support. A similar pattern is observed in localities 16 and 17, which are recovered as consecutive sister groups to the remaining Northeastern clade. Within the Northeastern clade, three well‐supported lineages were identified: Central Pyrenees, East Iberia, and Europe. The Central Pyrenees clade includes localities from the Aran Valley (localities 21–25), whereas the Eastern Iberia clade, distributed across Northeastern Catalonia, shows further substructure. The European clade comprises all populations from France, Belgium, and Germany, forming a monophyletic group suggestive of a single, recent recolonization of Europe. This European group is sister to samples from a single locality near Andorra (locality 28), suggesting the origin of the European recolonization route may lie in this region.

**FIGURE 3:**
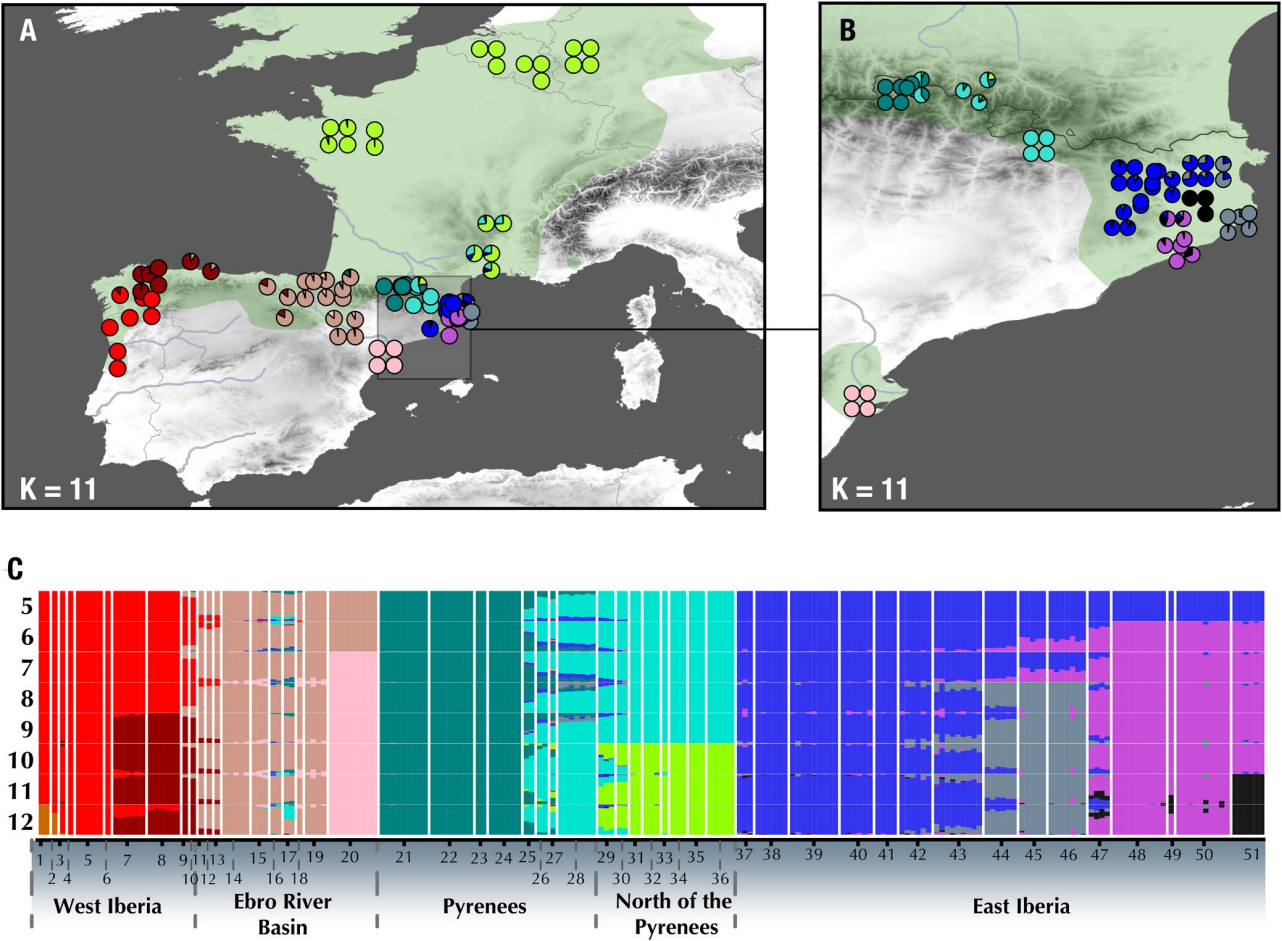
Population structure of *Lissotriton helveticus* . (A and B) Distribution maps showing the best‐k ADMIXTURE scenario separating *Lissotriton helveticus* into 11 genetic clusters. Pie graphs display admixture proportions for each individual. (C) ADMIXTURE plot showing the ancestry proportions for each genotyped specimen from *K* = 5 to *K* = 12. The best *K* configuration, as inferred by the minima across cross‐validation (CV) errors, is *K* = 11, although *K* = 9 and 10 also presented some of the lowest CV values. The current distribution of *L. helveticus* adapted from the IUCN Red List of Threatened Species (www.iucnredlist.org) is shown in green.

The multispecies coalescent time‐calibrated species tree presented a discordant topology from the ML tree (Figure 3). Mainly, the Ebro River Basin clade was recovered as sister to the Northeastern clade (divergence: 0.74 Ma), instead of grouping with the West Iberian populations. In this analysis, admixed individuals (see population structure results below) were discarded; therefore, those from localities 11, 12, and 13 (closer to the West Iberian populations) and from localities 16 and 17 (closer to the Northeastern clade) were not used to infer the species tree. The oldest split within *L. helveticus* dates back to around 1.1 Ma, separating West Iberian populations from the rest. Within the Ebro River Basin, populations follow a geographic cline, with those closer to the delta (locality 19) being recovered as sister to those found in the Ebro River Delta (locality 20).

Within the Northeastern clade, the earliest divergence occurred approximately 0.51 Ma and split the Central Pyrenean population located in the Aran Valley (localities 22 and 24) from the rest of the group. Within the latter, two main clades emerge: one containing all East Iberian populations in northeastern Catalonia and another composed of all European populations plus one population in the Pyrenees surrounding Andorra (locality 28). The Eastern Iberia clade has a crown age of 93,000 years. In contrast, the split between the Pyrenean and European populations is older, approximately 347,000 years ago (95% HPDi = 150,000–561,000 years ago), and all European populations present a crown age of 154,000 years ago (95% HPDi = 58,900–242,100 years ago). These results suggest that a single recolonization event of Europe took place between 560,000 and 58,900 years ago, in the Mid‐Late Pleistocene.

The phylogeographic reconstruction of *Lissotriton helveticus* suggests that the most recent common ancestor of all extant *L. helveticus* was likely distributed across northwestern Iberia (Figure 3). From there, it would have dispersed along a west–east corridor involving two main independent routes: one across the Ebro River Basin, and a second through the Pyrenees. From the latter, probably in the surroundings of Andorra, one clade dispersed eastward, establishing in northeastern Catalonia, whereas another crossed the Pyrenees and widely dispersed across western and central Europe.

### 3.2 Genetic Population Structure

**FIGURE 4:**
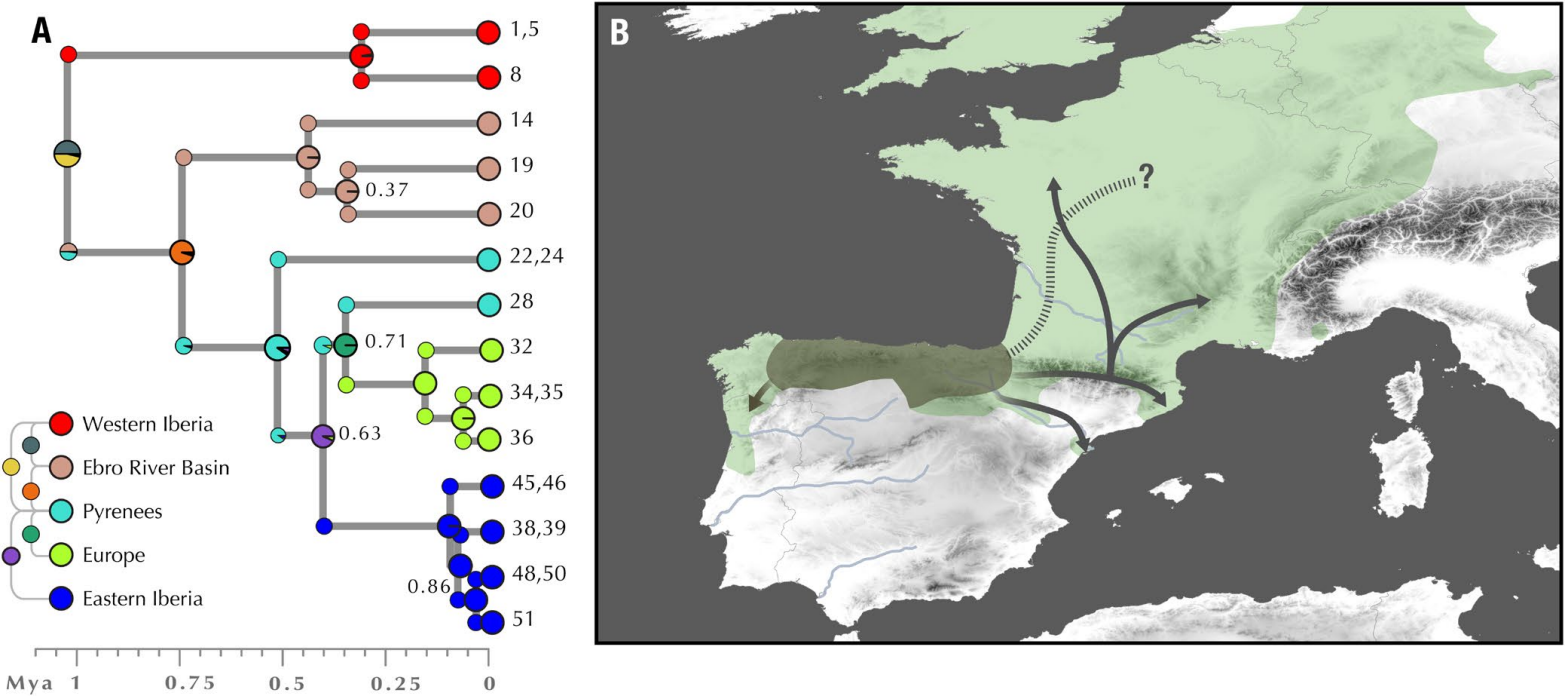
Biogeographic history of *Lissotriton helveticus* . (A) Ancestral range reconstruction of *L. helveticus* displayed on a time‐calibrated population tree for the species. Tips are color‐coded on the basis of five geographic regions displayed in the legend as well as two‐region scenarios. Node posterior probabilities are shown at each node when lower than 0.95. (B) Sketch of the biogeographic history of *L. helveticus* . The dashed line represents ancient dispersal into Iberia from a hypothetical ancestral area in central Europe. The area delimited in dark gray represents the most plausible glacial Iberian refugium, where the species would have persisted after the generalized cooling climatic regime of the early Pleistocene. Solid arrows show the subsequent Quaternary dispersal routes for the species. The current distribution of *L. helveticus* adapted from the IUCN Red List of Threatened Species (www.iucnredlist.org) is shown in green.

We explored the population structure of *Lissotriton helveticus* with a dataset of 205 specimens and 3603 unlinked SNPs each present in at least 60% of all individuals. The first two components of the PCA explained 29.1% of the variance in the dataset and primarily separated *L. helveticus* into four geographical clusters (Figure 1D): West Iberia, Ebro River Basin, East Iberia, and the Pyrenees (including all European localities). Interestingly, localities close to the contact zone between the West Iberia and the Ebro River Basin groups present intermediate cluster assignment probabilities (e.g., localities 9 and 10 in the West Iberia cluster or localities 11, 12, and 13 in the Ebro River Basin cluster), suggesting ongoing gene flow across clusters. Additionally, within the Ebro River Basin cluster, the specimens from the Ebro River Delta were retrieved as a distinct unit. The third principal component explained 7.8% of the variance, further separating the Central Pyrenees localities in the Aran Valley (localities 21–25) from those surrounding Andorra and across Europe (localities 26–36). Admixture analysis supported between 9 to 11 populations (Figure 4), with the minima across cross‐validation scores at *K* = 11. This configuration geographically separates all genetic clusters found in the PCA analysis, while also revealing finer‐scale geographical structure. Nearby localities generally exhibit notable levels of admixture (e.g., localities 10–14 in the contact zone between West Iberia and the Ebro River Basin, or localities 25–27 in the contact zone between the Central Pyrenees and the localities surrounding Andorra). Within the Ebro River Basin, locality 20 (Ebro River Delta) was recovered as an independent and isolated cluster from *K* = 7 onward, reflecting the isolation of this population. Notably, European localities remain grouped with the Pyrenean localities surrounding Andorra (localities 26–28) from *K* = 5 to *K* = 9, after which they are recovered as an independent population with admixed specimens in localities 29 and 30 (Figure 4).

**FIGURE 5:**
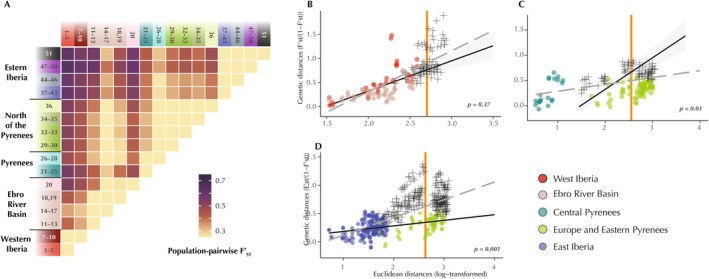
Genetic differences between *Lissotriton helveticus* populations. (A) Heatmap of population‐pairwise standardized fixation indexes (F'_ST_) among *L. helveticus* populations. Localities were assigned to the 11 genetic clusters retrieved from population structure analyses, with the exception of populations from the Ebro River Basin and Europe, which were further subdivided because of their distinct signs of admixture and geographic subdivision, respectively. (B–D) Relationships between genomic (calculated as F'_ST_/(1‐F'_ST_)) and log‐transformed Euclidean geographical distances (calculated in kilometers) in pairs of populations of *L. helveticus* lineages to explore isolation‐by‐distance (IBD) patterns. Colored circles: Distances between pairs of localities belonging to the same population. Black crosses: Distances between localities belonging to distinct populations. Black solid line: Regression line fitted only for within‐lineages comparisons and its 95% CI in light gray. Dashed‐gray line: Regression line fitted for all comparisons, either within‐ or between‐lineages (circles and crosses). In the bottom‐right corner, the *p*‐value of a Jackknife‐based test for equality of regression involving all distances (dashed‐gray line) and regression involving within‐group distances only (Black solid line). A significant *p*‐value supports those differences between lineages cannot be solely explained by IBD. Orange vertical line: Center of between‐lineages geographical distances.

Patterns of population‐pairwise F'_ST_ generally coincide with the phylogenetic signal, with the most genetically differentiated populations corresponding to the oldest intraspecific split, between West Iberian populations and all other lineages (Figure 5A). Each of the five main genetic clusters identified in the ADMIXTURE analysis exhibits lower F'_ST_ values among populations within the cluster, with some exceptions. For instance, the Ebro River Delta population (locality 20) displays higher F'_ST_ values with all populations than any other population in the Ebro River Basin lineage. This increased allele fixation could indicate long‐term isolation and/or small historical and current population sizes. In contrast, lower F'_ST_ values are observed between localities 14–17 and the European (localities 29–36) and Pyrenean localities surrounding Andorra (26–28), suggesting gene flow between these groups. This is consistent with ADMIXTURE results (Figure 4), where some individuals from localities 15, 16, and 17 display shared ancestry with Pyrenean populations. Finally, also in agreement with the results of previously described analyses, the Pyrenean localities surrounding Andorra (26–28) are recovered together with the European group, suggesting close genetic relationships between both groups.

Although IBD was found within all lineages, IBD alone could not account for the entire genomic differences observed between the Central Pyrenees and the European lineage—including the Pyrenean localities surrounding Andorra–(*p* < 0.01), or between the European and the East Iberia lineage (*p* < 0.001). Contrastingly, the genomic differences observed between Western Iberia and Ebro River Basin lineages can be solely explained by the distance between all its populations (*p* = 0.367), suggesting that they are a single unit differentiated along a geographic gradient (Figure 5B and Table S2). The structure within each *L. helveticus* lineage is concordant with a similar IBD pattern across lineages, as shown by their non‐significant intercept and slope (Table S2).

### 3.3 Demographic History

**FIGURE 6:**
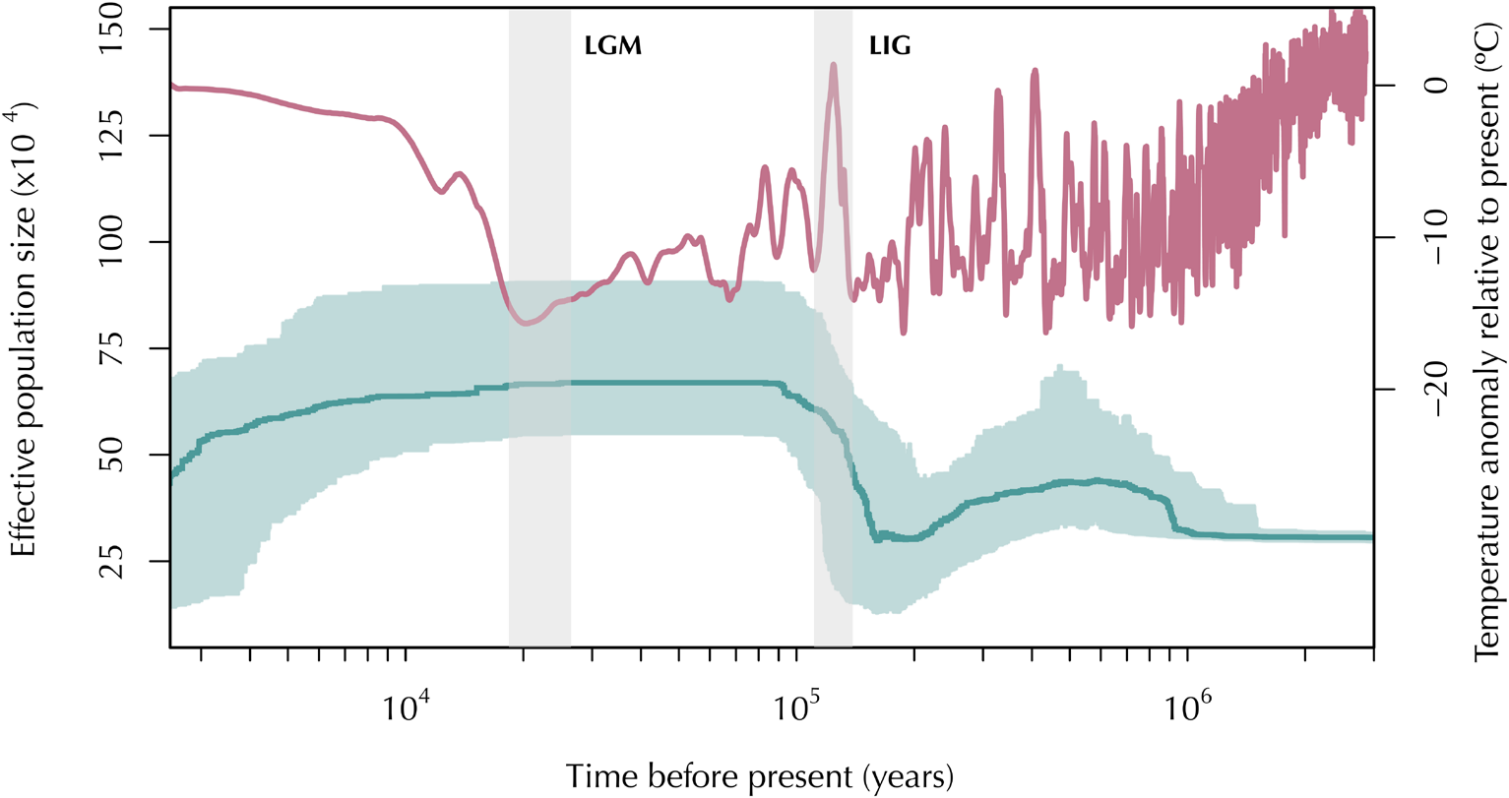
Demographic inference of historical population sizes in *Lissotriton helveticus* over the last 10^6^ years. The turquoise‐colored line represents the median effective population size (N_e_ × 10^4^) calculated with Stairwayplot2, with its corresponding 95% CI shaded in pale turquoise. The red line displays the Northern Hemisphere mean temperatures (de Boer et al. 2014) shown as temperature anomaly relative to the present. In gray, the Last Glacial Maximum (LGM) and the Last Interglacial (LIG) are highlighted. In the *x* axis, time before present is shown in a logarithmic scale.

Increases in Northern Hemisphere temperature anomalies over the Late Pleistocene and Holocene were significantly associated with historical increases in effective population size (*R* ^2^ = 0.40; *Z* = 6.4; *p*‐value < 0.001). No temporal autocorrelation was detected. The demographic history of *L. helveticus* is characterized by an initial period of stability until 1 Ma (~300,000 individuals), followed by a slight increase up to approximately 450,000 individuals, matching the first intraspecific divergence at 1 Ma (Figure 6). From then, effective population sizes followed a shallow and slow‐paced decrease from 400,000 to 160,000 years ago, reaching a minimum of 298,000 individuals. On the onset of the Last Interglacial Period (130–115 kya), effective population sizes dramatically rose, following global temperature increases, and stabilized at around 700,000 individuals. Since the end of the LGM (19 kya), effective population sizes have been slowly decreasing.

### 3.4 ENM

The ENM analysis revealed that Bio14 (Precipitation of the Driest Month) and Bio1 (Annual Mean Temperature) were the most important predictors of habitat suitability for *L. helveticus* , contributing 72% and 57%, respectively. The remaining variables, Bio12 (Annual Precipitation) and Bio15 (Precipitation Seasonality), contributed less than 30%, indicating a lesser impact on model predictions. The response curves for each variable provided insights into the relationship between environmental conditions and the probability of species presence. For Bio1, habitat suitability increased positively with temperature, showing a steep rise above 10°C and reaching a plateau at around 15°C. In contrast, Bio14 displayed a unimodal response, with suitability peaking around 60 mm of precipitation. Lower and higher precipitation values resulted in a decline in habitat suitability, forming a bell‐shaped curve. Among the remaining predictors, Bio12 showed a gradual positive response, with suitability increasing as annual precipitation levels rose. Meanwhile, Bio15 exhibited a negative relationship with suitability. Model evaluation metrics confirmed the robustness of the predictions, with high sensitivity (true positive rates) and specificity (true negative rates) achieved by both the TSS‐based ensemble model and the ROC‐based evaluation model (Table S3).

**FIGURE 7:**
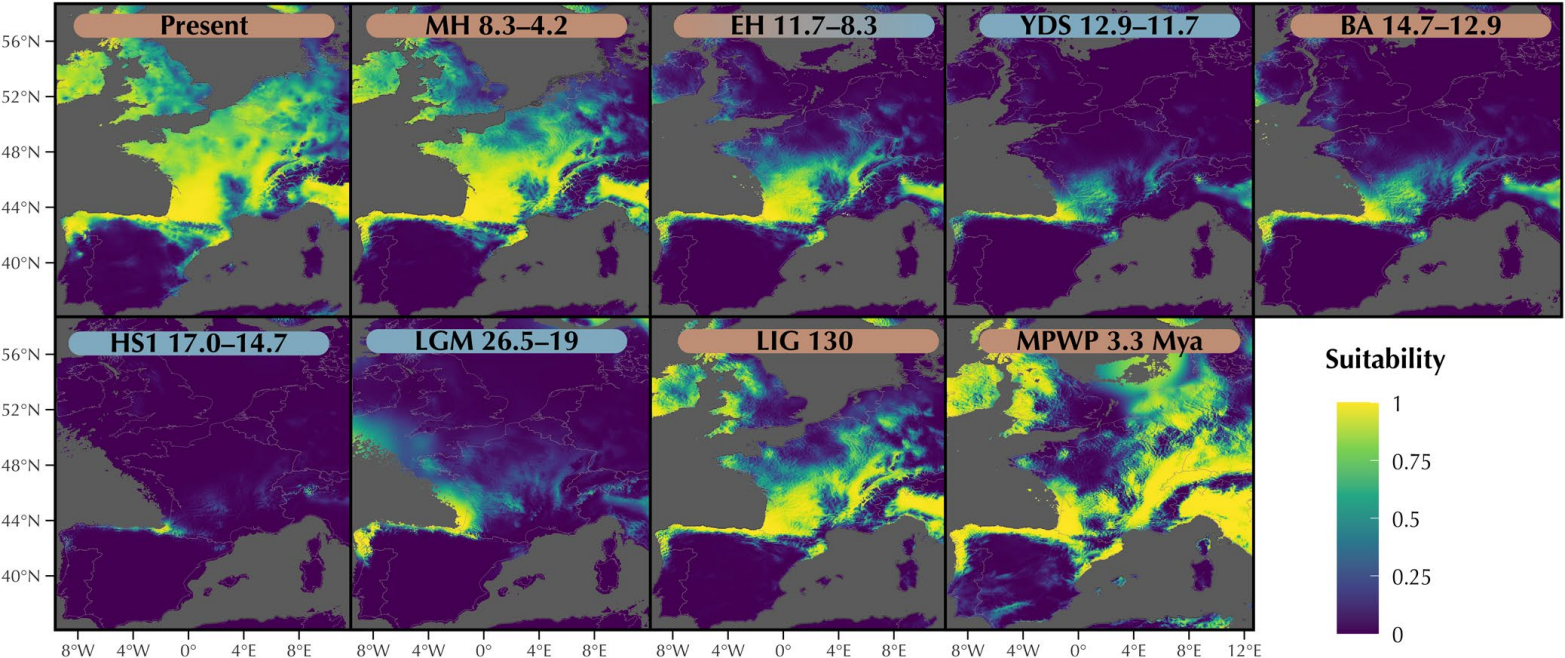
Ecological Niche Modeling of suitable habitat for *Lissotriton helveticus* predicted with a GLM model and projected into different paleoclimatic periods: BA, Bølling‐Allerød; EHG, Early‐Holocene, Greenlandian; HS1, Heinrich Stadial 1; LGM, Last Glacial Maximum; LIG, Last Interglacial; MH, Mid‐Holocene, Northgrippian; MPWP, Mid‐Pliocene Warm Period; YDS, Younger Dryas Stadial. All paleoclimatic layers were downloaded from Paleoclim (http://www.paleoclim.org). Brown and Blue labels correspond to warm and cold periods, respectively. EH represents a transition period. Numbers represent thousands of years (except for MPWP, in millions of years).

Projections of the predicted models over the study area under current climatic conditions accurately displayed the known distribution of *L. helveticus* . When projected onto past climatic reconstructions, suitable areas for *L. helveticus* decreased during cold periods and expanded during warmer periods. During the Mid‐Pliocene Warm Period (MPWP; 3.3 Ma) and the Last Interglacial (LIG; 130 kya), suitable habitat for *L. helveticus* encompassed an area similar to its current distribution, including large areas in Europe. However, the drastic temperature drops of the LGM (26.5–19 kya) and the Heinrich Stadial 1 (HS1; 17–14.7 kya) substantially reduced the extent of suitable areas for *L. helveticus* , confining it to the Northern Iberian Peninsula. The subsequent gradual increase in global temperatures (with the exception of the cold period over the Younger Dryas Stadial) was accompanied by a corresponding expansion in habitat availability for the species. The species spread northward from the Pyrenees into the Aquitaine Basin in southern France, and further north and east, eventually reaching the full extent of its current distribution (Figure 7).

## 4 Discussion

In this study, we reconstructed the evolutionary history of *Lissotriton helveticus* by generating a comprehensive genomic dataset for 205 specimens sampled across its distribution range. Our results provide new insights into the diversification and historical population dynamics of *L. helveticus* , shaped by Quaternary climatic oscillations.

### 4.1 *Lissotriton* Diversification Dynamics

Our phylogenomic reconstruction (Figure 2) supports a sister group relationship between *Lissotriton helveticus* and *L. vulgaris* , which would have diverged approximately 25.5 Ma. These results are in agreement with recent phylogenomic studies (Mars et al. 2025) but contrast with previous phylogenies reconstructed from a few mitochondrial and nuclear markers (Pabijan et al. 2015; Recuero et al. 2014). Furthermore, our results place *L. italicus* at the deepest split in the genus, sister to the remaining taxa analyzed, whereas *L. boscai* would have diverged from the *L. helveticus‐vulgaris* lineage approximately 32.7 Ma. The divergence of *L. helveticus* and *L. vulgaris* aligns with major climatic transitions, particularly the cooling period at the Eocene–Oligocene boundary (Liu et al. 2009), which might have fragmented ancestral distributions promoting diversification processes in the genus.

Phylogenomic reconstructions of intraspecific diversification in *L. helveticus* support extremely recent diversification events at 1.1 Ma, contrasting with the ancient origin of the species (Figure 3). The lack of deep genomic lineages in Iberia is congruent with the “refuge” model hypothesis, possibly exacerbated by significant population bottlenecks at the onset of Quaternary glacial cycles. Such observed patterns could be explained by a Central European origin of *L. helveticus* , with subsequent colonization of Iberia driven by generalized global cooling periods, and ultimately becoming extinct from most of its original distribution (Recuero and García‐París 2011). The presence of *Lissotriton helveticus* in central Europe over warmer periods is also supported by our projected paleoniche reconstructions such as the Mid Pliocene paleoniche reconstruction at 3.3 Ma, which suggests that large parts of Europe were suitable for the species (Figure 7).

Despite its relatively recent diversification, population structure analyses identified five well‐defined geographically separated groups within *L*. *helveticus* : West Iberia, Ebro River Basin, Central Pyrenees, East Iberia, and North of the Pyrenees (including all European populations and some localities in the Eastern Pyrenees). Genetic differences between West Iberia and the Ebro River Basin lineages can be explained solely by IBD (Figure 5; Table S2), which suggests that both populations evolved under the same IBD gradient and thus represent a single continuous population. Therefore, further analysis in the contact zone between these groups should be conducted, including a wider sampling, to discern if the observed population structure is an artifact produced by the sampling gap between both clusters rather than a true genetic discontinuity. In contrast, the other lineages exhibit a strong genetic structure, which cannot be explained by IBD alone and matching geographic barriers and historical refugia. However, some admixture is evident in areas of transition across genetic groups, indicating ongoing gene flow (Figure 4). For instance, the contact zone between the Central Pyrenees and the populations around Andorra shows admixture despite their relatively deep divergence. Additional secondary contact zones appear to be forming between Western Pyrenees (localities 15–17) and both European (localities 29–36) and Pyrenean localities surrounding Andorra (localities 26–28), as evidenced by low F'_ST_ estimates among populations (Figure 5A). Given that Western Pyrenean populations are more genetically differentiated from Central Pyrenean localities (localities 21–25) than from European ones, and that a continuous corridor of suitable habitat between southeastern France and northern Iberia has existed at least over the last 14,000 years (Figure 7), the most plausible explanation for this pattern involves gene exchange with European populations backcrossing the mountain range through the lower elevations in the westernmost side of the Pyrenees. Altogether, these results highlight the role of the Pyrenees as a major, dynamic barrier to gene flow in *L. helveticus* , likely isolating populations during glacial periods but becoming more permeable during interglacials.

The current ecological niche of *L. helveticus* suggests that no major geographic barriers exist across its distribution in the Iberian Peninsula (Figure 7). Therefore, the pronounced genetic differentiation observed across *L. helveticus* lineages, combined with evidence of admixture at lineage boundaries, suggests that the observed structure primarily reflects the cyclical patterns of isolation and dispersal with secondary contacts imposed by Quaternary glacial oscillations. During glacial maxima, populations likely became isolated within climatically stable refugia, promoting lineage divergence, whereas subsequent interglacial periods facilitated range expansions and secondary contacts, leading to the current admixture patterns (Figure 4). Although such secondary contacts might be masking the phylogenetic signal and contributing to the discrepancies between ML and BI phylogenies (Figures 2 and 3), incomplete lineage sorting could also be a significant factor in phylogenomic incongruence, especially given the recent divergence of *L. helveticus* lineages.

### 4.2 Phylogeographic History

Phylogeographic reconstructions set the origin of the current diversity of *L. helveticus* along northern Iberia, between the headwaters of the Ebro River and the Cantabrian Range (Figure 3). These results align with previous studies (Recuero and García‐París 2011) and are consistent with patterns observed in other temperate taxa, such as *Vipera seoanei*, which also survived Quaternary climatic oscillations in North Iberian refugia (Martínez‐Freiría et al. 2015). This long‐term persistence is further supported by the projected paleoniches, which indicate that during colder periods, suitable habitats for *L. helveticus* were largely confined to northern Iberia (Figure 7). Although these reconstructions extend only as far back as the LGM, similar habitat restrictions likely occurred during earlier glacial events for which data were not available.

The first divergence within the species occurred approximately 1.1 Ma, splitting the West Iberia cluster from all other *L. helveticus* . Since differences between the West Iberian and the Ebro River Basin clusters can be solely explained by geographic distance, they likely represent a single, contiguous, and widespread lineage that simultaneously expanded westward and eastward from its original refugium in northern Iberia. The Cantabrian Range seems to have acted as a functional corridor for this species, as no evident genetic breaks between localities were observed. Within this population, one group dispersed across the Ebro River Basin, continuously expanding its distribution following water bodies along the river edges until reaching the Ebro River Delta. The increased humidity and vegetation cover of primary and secondary river basins along the Ebro River probably ameliorated climatic conditions, favoring the dispersal of *L. helveticus* , especially during cold periods. One interesting case is the population from the Ebro River Delta (locality 20). This population, located in a small range near the delta (Montsià Range), was introduced during the 1960s from a now‐extinct natural population from the right margin of the Ebro River's predeltaic plain in Amposta (Rivera et al. 1994). Given the significant distance from the nearest known localities (~250 km), the origin of the predeltaic populations had long been uncertain, with some authors hypothesizing that they were established via a dispersal event along the Ebro River (García‐Salmerón et al. 2022; Llorente et al. 1991; Recuero and García‐París 2011), though this had never been formally demonstrated. Our phylogenomic reconstructions and population structure analyses now confirm this hypothesis, providing clear evidence that the Ebro River Delta population is not closely related to any other Catalonian populations. Locality 20 appears nested within the Ebro River Basin lineage, showing traces of admixture with other populations along the basin (Figure 4) and exhibiting signs of long‐term isolation or small population sizes, as evidenced by the F'_ST_ results (Figure 5A). The estimated divergence with its nearest relative (c. 350 kya) suggests an old dispersal along the Ebro River, emphasizing the importance of waterways as key vectors for long‐distance dispersal in this species. Altogether, we confirm that this population actually represents a relic lineage markedly distinct from all other *Lissotriton* in Catalonia, which has important implications for conservation management.

Although *L. helveticus* was historically abundant along the margins of the Ebro River, it is now very rare—or even completely absent—from the middle and lower reaches of the river (Campo Giménez and Ruiz Ara 2019; García‐Salmerón et al. 2022; Llorente et al. 1991; Pleguezuelos et al. 2002; Roig 2008). This decline is primarily due to habitat loss, pesticide use, and the introduction of invasive species such as the red swamp crayfish ( *Procambarus clarkii* ), which voraciously preys on both larvae and adults (Roig 2008). Furthermore, increasing contemporary threats, such as the recent introduction of the Blue Crab ( *Callinectes sapidus* ; Castejón and Guerao 2013), or the American Bullfrog (*Aquarana catesbeiana*; Sanz et al. 2023) could further hamper the recolonization of the species' natural range, as these invasive species now inhabit areas where *L. helveticus* formerly occurred along the delta and predeltaic plains. Paradoxically, the introduction of locality 20 into the Montsià Range in the 1960s may have helped preserve this lineage and prevented its extinction. Therefore, effective management strategies should be implemented to ensure the survival of the only representative population of the Ebro River Basin lineage in Catalonia.

On the other hand, a second group dispersed from North Iberia through the Pyrenees, migrating eastward and northward. Given the complex topography of this mountain range, with peaks reaching over 3000 m, populations most likely survived glacial periods in lower‐elevation refugia along the Pre‐Pyrenean mountains, as in other amphibians and reptiles like the genus *Calotriton* (Carranza and Amat 2005; Talavera et al. 2024) or *Iberolacerta* species in the subgenus *Pyrenesaura* (Carranza et al. 2004; Talavera, Burriel‐Carranza, et al. 2025). The complex structure found between geographically close localities (i.e., localities 21–25 vs. 26–28) exemplifies such isolation and independent diversification between lineages, despite their current geographic proximity. Approximately 400 kya, one Pyrenean clade reached the easternmost side of the mountain range and established populations along the littoral and pre‐littoral ranges facing the Mediterranean Sea (populations 37–50). The long branch connecting this clade and its sister group (Figure 3) suggests that some intermediate populations may have gone extinct. Most likely, the proximity of these populations to a large water body enabled their survival through glacial periods in isolated refugia along areas that remained suitable for the species even during the LGM (Figure 7).

The Pyrenean clade established populations along the Eastern Pyrenees and set the first step toward Europe's recolonization. The origin of the out‐of‐Iberia migration route has been a subject of debate, as the extremely recent dispersal and low genetic diversity of populations in the recolonized range make the reconstruction of possible pathways extremely challenging (Elfering et al. 2024; Recuero and García‐París 2011). However, our genome‐wide SNP dataset allowed us to pinpoint the source populations to a specific region in the Pyrenees surrounding Andorra, encompassing localities 26–28. Both phylogenomic reconstructions (Figures 2 and 3) and population genomic analyses (Figures 4 and 5) consistently identified these populations as the source of the European genetic pool. Notably, the area encompassed by these populations corresponds to the headwaters of the Ariège River, a tributary of the Garonne River, the major river in southwestern France, which flows through Toulouse and ultimately reaches the Atlantic Ocean near Bordeaux.

According to our phylogeographic reconstructions, these Pyrenean populations started dispersing northward as early as 345,000 years ago. Further diversification, however, did not occur until c. 150,000 years ago, broadly coinciding with the Last Interglacial period (Figure 3). Thus, the two‐fold increase in effective population sizes observed during the Last Interglacial period (Figure 6) likely reflects range expansions of populations into suddenly available habitat. Furthermore, although strong population bottlenecks likely occurred during the LGM (c. 21,000 years ago), suitable areas persisted near the Garonne River (Figure 7), which probably allowed the survival of already established European populations in this refugium. These results contrast with previous hypotheses suggesting a Holocene recolonization of Europe (Recuero and García‐París 2011) and suggest that populations north of the Pyrenees were probably more resilient to the extreme climatic conditions during glacial periods than previously thought. The importance of waterways in dispersal and evolutionary dynamics in amphibians is further illustrated by the Garonne River, which appears to have acted as both a conduit for long‐distance dispersal and a climatic refugium in multiple species, including *Lissotriton helveticus* , *Salamandra salamandra* (García‐París et al. 1998; Burgon et al. 2021; Gippner et al. 2024), *Bufo spinosus* (Arntzen et al. 2017), *Pelodytes punctatus* (Díaz‐Rodríguez et al. 2015), or *Pelobates cultripes* (Gutiérrez‐Rodríguez et al. 2017).

## 5 Conclusions

In this study, we assembled a comprehensive genomic dataset comprising 205 *Lissotriton helveticus* individuals spanning the species' entire distribution range. Combined with projected paleoniche reconstructions, the genomic data gathered herein shed light on the complex evolutionary and phylogeographic history of recently differentiated intraspecific lineages in the species. Our findings highlight the role of Quaternary climatic oscillations in shaping the current distribution and genetic structure of *L. helveticus* . From its original refugium in northern Iberia, this temperate species underwent cyclic processes of dispersal, isolation, and subsequent range expansions, followed by secondary contacts, resulting in complex population structure marked by differentiation and admixture. Ancestral state reconstructions suggest two main dispersal routes out of the original refugium in northern Iberia: (i) eastward along the Ebro River Basin and (ii) eastward into the Pyrenees and subsequently northward into Europe. Our results highlight the role of rivers as key drivers of historical dispersal dynamics in amphibians, serving as corridors for long‐distance dispersal and effectively acting as refugia during glacial periods. Moreover, the increased resolution provided by genome‐wide nuclear markers enabled us to precisely pinpoint the geographical origin of the out‐of‐Iberia recolonization route to a specific region in the Pyrenees surrounding Andorra and its adjacent valleys during the Mid‐Late Pleistocene. Despite experiencing severe bottlenecks during glacial periods, stable populations seem to have persisted in climatically favorable areas north of the Pyrenees, as supported by both ancestral state reconstructions and paleoniche modeling, in line with an increasingly important role for extra‐Iberian refugia shaping current biodiversity.

## Author Contributions

**Bernat Burriel‐Carranza:** conceptualization (lead), data curation (equal), formal analysis (equal), investigation (equal), methodology (equal), project administration (equal), resources (equal), software (equal), supervision (equal), validation (equal), visualization (equal), writing – original draft (equal), writing – review and editing (equal). **Jhulyana López‐Caro:** data curation (equal), formal analysis (equal), investigation (equal), methodology (equal), writing – review and editing (equal). **Adrià Jordà:** data curation (equal), formal analysis (equal), investigation (equal), methodology (equal), writing – review and editing (equal). **Loukia Spilani:** formal analysis (equal), writing – review and editing (equal). **Adrián Talavera:** formal analysis (equal), writing – review and editing (equal). **Gabriel Mochales‐Riaño:** writing – review and editing (equal). **Martiño Cabana:** resources (equal), writing – review and editing (equal). **Albert Montori:** resources (equal), supervision (equal), writing – review and editing (equal). **Pierre‐André Crochet:** resources (equal), writing – review and editing (equal). **Ernesto Recuero:** resources (equal), writing – review and editing (equal). **Mario García‐París:** resources (equal), writing – review and editing (equal). **Íñigo Martínez‐Solano:** resources (equal), writing – review and editing (equal). **Daniel Fernandez‐Guiberteau:** conceptualization (equal), funding acquisition (equal), resources (equal), supervision (equal), writing – review and editing (equal). **Salvador Carranza:** conceptualization (equal), funding acquisition (equal), project administration (equal), resources (equal), supervision (equal), validation (equal), writing – review and editing (equal).

## Conflicts of Interest

The authors declare no conflicts of interest.

## Supporting information


**Table S1:** ece371994‐sup‐0001‐Tables.zip.
**Table S2:** ece371994‐sup‐0001‐Tables.zip.
**Table S3:** ece371994‐sup‐0001‐Tables.zip.

## Data Availability

Raw demultiplexed ddRAD sequence reads used in the present study have been deposited in the sequence read archives (SRA) under the Bioproject name PRJNA1241519. Benefits Generated: Benefits from this research accrue from the sharing of our data and results on public databases as described above.
